# Rebirth of Distributed AI—A Review of eHealth Research

**DOI:** 10.3390/s21154999

**Published:** 2021-07-23

**Authors:** Manzoor Ahmed Khan, Najla Alkaabi

**Affiliations:** College of Information Technology, United Arab Emirates University, Al Ain, Abu Dhabi 15551, United Arab Emirates; 960223676@uaeu.ac.ae

**Keywords:** federated learning, eHealth, data privacy, distributed computing

## Abstract

The envisioned smart city domains are expected to rely heavily on artificial intelligence and machine learning (ML) approaches for their operations, where the basic ingredient is data. Privacy of the data and training time have been major roadblocks to achieving the specific goals of each application domain. Policy makers, the research community, and the industrial sector have been putting their efforts into addressing these issues. Federated learning, with its distributed and local training approach, stands out as a potential solution to these challenges. In this article, we discuss the potential interplay of different technologies and AI for achieving the required features of future smart city services. Having discussed a few use-cases for future eHealth, we list design goals and technical requirements of the enabling technologies. The paper confines its focus on federated learning. After providing the tutorial on federated learning, we analyze the Federated Learning research literature. We also highlight the challenges. A solution sketch and high-level research directions may be instrumental in addressing the challenges.

## 1. Introduction

Smart cities aim at providing robust solutions to crucial societal challenges related to transportation, health, environment, education, and security [1]. Smart cities are expected to deploy massive Internet-of-Things (IoT) related devices and applications. It is estimated that 75 billion devices will be connected by 2025, a jump from 31 billion in 2020 [2]. Moreover, the amount of data generated from devices or humans is growing every day. According to estimates, 4 TB of data will be generated by the self-driving car per day [3] and the amount of data created over the next three years will be more than the data created over the past 30 years [4]. With this explosion of big data and the proliferation of cheaper sensors and mobile devices, along with the advancement in both wireless-communication and deep learning, we envision a wide spectrum of different applications that would revolutionize many industries, businesses, services, and our day-to-day lives. These applications will be characterized by being data-driven and distributed.

Learning from the data, in the case of the smart city operations, enables the stakeholders to reinforce and optimize the performance of various smart city operations. However, a larger portion of such data is usually generated by users who are sensitive to privacy infringement [5]. Even if the data privacy concern is addressed, yet another challenge remains, which is sending the massive volumes of data to a central server that may heavily congest the communication bit-pipes [6]. Furthermore, another pain point of AI’s applications in smart cities is the training time of the ML model. Even with the availability of massive computing power and the evolution of deep neural networks, it is impossible to perform the most complex analyses without going through the time consuming phase of pre-processing and feature selection.

To tackle these and similar challenges, the concept of distributed learning turns out to be a promising solution. It will not only assist in mitigating the congestion on the communication bit-pipes but also enable the implementation of collaborative models without compromising the privacy of the data.

Achieving the required features for the envisioned smart city applications require a paradigm shift in the current technologies. We are already witnessing a shift from centralized data-computing, whether on-premises or in the cloud, to a distributed data-computing, namely, edge computing. As per [7], edge computing will not substitute the cloud, but its adoption is definitely growing. The need of edge computing is inevitable with the pervasiveness of the IoT and the increase in data generated, especially the ephemeral data that should not be transferred to the cloud. Moreover, edge computing is necessary due to the need for real-time and on-spot processing such as in life-critical real-time health monitoring applications.

Federated Learning (FL) addresses the aforementioned and similar problems [8]. It allows stakeholders to train the model with their local datasets without violating their privacy. The stakeholders then share the hyper parameters or gradients of their locally trained model with the orchestrating central server [9]. The server keeps updating the central model through captured hyper parameter values from the clients. This is to say that each client feeds in to the global model at the central server and then downloads the updated global model. The central server keeps looping this process until the learning is matured [10]. The recent concept of Mobile Edge Computing (MEC) has further added to the impact of federated learning by enabling the placement of the central model nearer to the device layer, which is an important achievement when it comes to realizing more complex use-case scenarios requiring active learning and real-time decisions for critical maneuvers.

Federated learning, distributed artificial intelligence, modular solutions, and service chaining are all based on distributed systems and are dynamically adaptable inter-entities/inter-stakeholder relationships, etc. that ask for efficient communication bit-pipes. Recently commercialized 5G mobile networks do pledge to meet service quality requirements of the present and near-future services. However, 5G may not be able to fulfill the demands of the future emerging intelligent and automation systems after 10 years. Therefore, not long after or even prior to the launch of 5G, the research to design the next wireless communication generation, namely the sixth generation (6G) system, has kicked off. It is expected that 6G will have the full support of artificial intelligence and satellite integration. Moreover, End-to-End latency requirements will also be met. An extremely low-latency feature would have a dramatic positive impact on real-time applications.

Among the three major enabling technologies involved in future applications, our focus in this survey is federated learning, specifically in future intelligent health applications. We chose to focus on federated learning due to its unique contribution in preserving privacy, which is a non-negotiable requirement for future intelligent healthcare applications. FL is a nascent field. It was introduced by Google in 2016. The topic is currently under exploration by researchers into FL’s capabilities, limitations, challenges, and potential enhancement.

Next, we discuss our perception of the future eHealth, which highlights the aforementioned interplay of the technologies.

Table 1 consolidates the major abbreviations and acronyms used in this paper.

## 2. Major Contributions of the Paper

In this paper, we focus on discussing the application and potential impact of federated learning in a major smart city domain, namely e-Health. Major contributions of the paper may be summarized as follows:

- Look into the eHealth of the future: Discussion on the envisioned eHealth services and applications, their design requirements, and the potential interplay of their enabling technologies.
- Analyses of the literature surveys about federated learning and related topics. This will direct the readers to the correct source.
- Federated learning overview: A tutorial section to equip readers with the background information that will enable them to comprehend all aspects of federated learning.
- Federated learning challenges: We pack the challenges under different categories, which assist in categorizing the solution approaches of the research literature.
- Literature survey of the FL solution approaches: An exhaustive survey of the research literature on FL solutions is carried out. To enhance the readability and analyze the impact of solution approaches in addressing the challenges, we map the solution approaches to the categories of challenges.
- Federated learning in a major application domain: driven by the expertise and research interests of the authors, we focus on one major application domain in this regard, i.e., eHealth.

**Figure 1:**
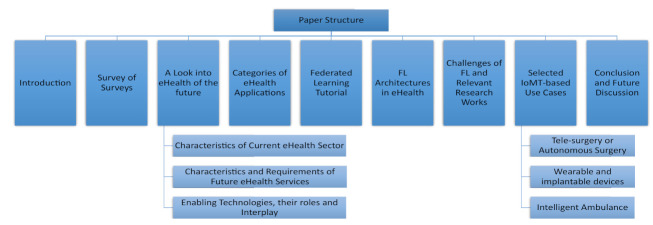
The structure of the paper.

Figure 1 shows structure of the paper, which depicts positioning of major contributions and relevant literature.

There have been a few review surveys on federated learning recently with or without a concentration on a specific industry or domain. For example, [11] gives a detailed survey about federated learning in mobile edge networks. It thoroughly reviews the different implementations and challenges. Specifically, the survey focuses on the challenges of communication costs, resource allocation, and privacy and security in the implementation of FL at scale. Another survey [12] gives an overview of technical details that pertain to FL enabling technologies, most recent platforms, protocols, and applications. It also summarizes the recent platforms such as PySyft, LEAF, and TFF in terms of their focus and supporting software. The research work in [13] goes further in describing these platforms by providing a categorization for federated learning platforms (or frameworks) according to six different aspects, namely data partition, machine learning model, privacy mechanism, communication architecture, scale of federation, and motivation of federation. Additionally, refs. [14,15,16] are other domain-agnostic surveys about federated learning that define FL, its different architectures, algorithms, challenges, and applications. We also find surveys about the integration of the futuristic 6G network with FL, such as in [14], where the authors describe key technical challenges of such integration. Although [17] is not an FL-exclusive survey, it dedicates a section on the role of FL in future wireless applications. Interestingly, it discusses in-depth the wireless AI applications in various data-driven domains. Other researchers surveyed sub-problems of federated learning. For example, we find [18] focuses on personalization techniques in FL, while [19] focuses on data poisoning attacks against FL systems.

It is common for most of domain-agnostic FL surveys to dedicate a section, with a varied level of depth, about the potential applications and use cases of FL in domains such as health, autonomous driving, or manufacturing. However, none of these map the applications of one domain to its possible FL implementation strategies. On the other hand, looking into the domain-specific FL surveys, we find few that concentrate on FL in vehicular Internet of Things [20,21] or Industrial IoT [22]. There are very few surveys that focus on FL in the healthcare sector exclusively. As far as we know, the existing surveys and reviews about FL in the healthcare sector are “Federated Learning for Healthcare Informatics” [23], which explains FL technology with a focus on Electronic Health Records (EHR) applications, and the second survey, “The future of digital health with federated learning” [24], which briefly explains the role of FL in health, again mainly in EHR applications. See Table 2.

## 3. A Look into eHealth of the Future

This section walks the reader through the characteristics of today’s eHealth sector, and the envisioned future eHealth services and applications. We aim in this section, after explaining the current baseline of characteristics and limitations, to understand the design requirements and the enabling technologies of future services including FL.

### 3.1. Characteristics of Current eHealth

As per Accenture [25], the AI health market is going through an explosive growth, as the market size is expected to reach US $6.6 B in 2021, compared to the US $600 M in 2014. What behind this rocketing increase in investment is the strong belief in the valuable insights that can be derived using AI to help improve the quality of the healthcare services, reduce complications, and enhance treatment outcomes. However, there are some hurdles that can curb the pervasive adoption of AI in healthcare sector.

Firstly, the healthcare sector is one of the most stringently regulated industries. Patient medical records, privacy, and security are of paramount importance. Although there are regulations and laws on how not to share data, there is not enough regulations on facilitating the sharing and exchanging of medical data. Moreover, the domain is characterized by the shortage of annotated training data, which is the key for building a good prediction model. Compared to other domains, the health sector has far fewer valuable public resources. Even if experts could gather and organize the records to produce some good training data, annotation in the health sector is not an easy task and is a time-consuming one. For instance, manual labeling of a single 3D brain MRI scan can take up to a week by a trained neuroanatomist [26].

What adds to the scarcity of resources is the limitation of learning transferability in this sector. It is prohibited to transfer pre-trained models out of a medical entity’s premise to another entity. Moreover, the volume of medical records in one entity is not large enough to train and produce a solid predictive model. All these factors slow the advancement of applying AI in the health sector as opposed to other domains.

### 3.2. Characteristics and Requirements of Future eHealth

Whether it is an application in healthcare, smart manufacturing, or autonomous driving, all future applications will be characterized by being data-driven. This means that data will dictate future services and how services would be designed, developed, personalized, improved and measured. Data is the new oil of the future, and insights driven out of raw data would be the key for advancement in industries at different levels. Whether it is historical data or real-time data, the envisioned services would make use of data to guide decisions about enhancing the Quality of Experience (QoE). The envisioned healthcare is expected to improve quality of life and to serve the mass of patients remotely and with a minimum intervention by humans, especially after the outbreak of COVID-19 and the fear of similar pandemics. Future services would ambitiously monitor the patient’s real-time health status (or the environmental factors around the patient) via a smart watch or implanted device that would proactively forecast health risks and respond to any worrying signs. Imagine emergency response before a heart attack gets started by forecasting its inception using the person’s vital signs [27]. The technical ability to sense, compute, communicate, train and infer ML models, and act on real time data are some of the technical requirements for the future eHealth services. In addition to those, services and applications must accommodate the non-technical requirements, namely the data privacy regulations. Processing data within the stringent bounds of privacy is one of the nonnegotiable requirements in eHealth. Medical data about patients must remain within the confined premise of the health institution. When the date are needed to be transmitted over the internet, they must be handled differently from what is conventional. In a nutshell, we can say that the success of these applications hinges on fulfilling three overarching requirements, hinted at above, and summarized as:

- Real-time intelligence
- Distributed intelligence
- Privacy-preserving enforcement

It is worth mentioning that not all future applications and services would require these three elements together. However, those that do would make the breakthrough in the quality of services and optimum personalized user experience.

### 3.3. Enabling Technologies, Their Roles and Interplay

The three aforementioned overarching requirements can be fulfilled by the interplay of three envisioned revolutionary technologies and concepts, namely:

- 6G mobile networks—enabling the needed communication bit-pipe requirements.
- Smart edges—bringing computation and intelligence nearer to the consumers of the services.
- Artificial intelligence—to implant intelligence, minimize human intervention, and enforce the privacy requirements through federated learning.

**Figure 2:**
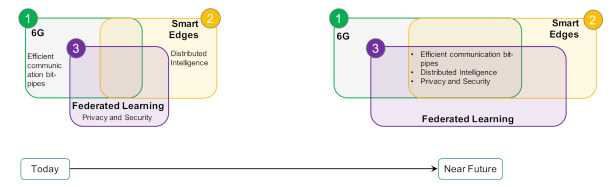
Envisioned Interplay of the enabling technologies and concepts.

Each technology will take the lead in fulfilling one requirement but still contributes to the other two. For example, 6G mobile networks will be responsible for minimizing latency and increasing the bandwidth of a communication channel to deliver real-time exchange of data, hence it will lead to fulfilling the real-time intelligence requirement. Nevertheless, 6G mobile networks will also contribute to the security element and distributed computation by enforcing network security and empowering IoT communication, for example. The interplay between the enabling technologies, namely federated learning, the 6th generation of mobile networks (6G), and smart edges, is pictorially depicted in Figure 2. The world is already witnessing the interplay of similar technologies in different application domains for their inherent features such as: efficient communication bit-pipes, ensuring privacy and security, and distributed decision making. The figure captures this by using a relatively smaller overlapping area covered by all the three technologies and concepts, which we expect will widen in the very near future (represented by the wider overlapping area).

#### 3.3.1. The Role of 6G

Compared to 4G, 5G networks are offering better services with significant advancement in terms of data rate, latency, and capacity. Although 5G has laid the foundation for supporting AI-empowered applications, there is still a need to fill the gaps in many of the requirements for full AI adoption and support. Therefore, research and development of the new wireless generation network, 6G, is actively ongoing. It is expected that in 2030, with the realization of the envisioned 6G, full AI-empowered health applications will be possible.

Table 3 reflects the enhancement 6G will bring compared to its predecessor 5G [28,29]. As the figures reflect, compared to 5G, 6G will be faster, more reliable, wider in coverage, and greater in bandwidth and capacity. It will meet all the communication requirements for future health applications of real-time and distributed intelligence. In terms of real-time intelligence, ultra-low latency is the key. With E2E latency that is less than 1 ms, 6G will revolutionize real-time health applications. In addition to ultra-low latency, 6G’s increased data rate of 1 Tbps, and the tripled bandwidth (compared to 5G), 6G will allow life-critical health applications such as telesurgery and intelligent ambulances to take a great leap forward. This increase is due to the envisioned utilization of THz transmission [30].

In terms of distributed intelligence, the 6G communication network will be ubiquitous and integrated and will provide deeper and broader coverage through device-to-device. Such coverage will be feasible by using terrestrial and satellite mobile communication. It is estimated that satellite transmission distance will reach above 600 km compared to 1 km terrestrial [31]. The increased capacity and mobility of 6G will also realize the ubiquitous intelligence by accommodating the connectivity of a large number of devices and sensors and through its ability to manage nodes mobility with proactive cashing [28].

#### 3.3.2. The Role of Edge Computing

**Figure 3:**
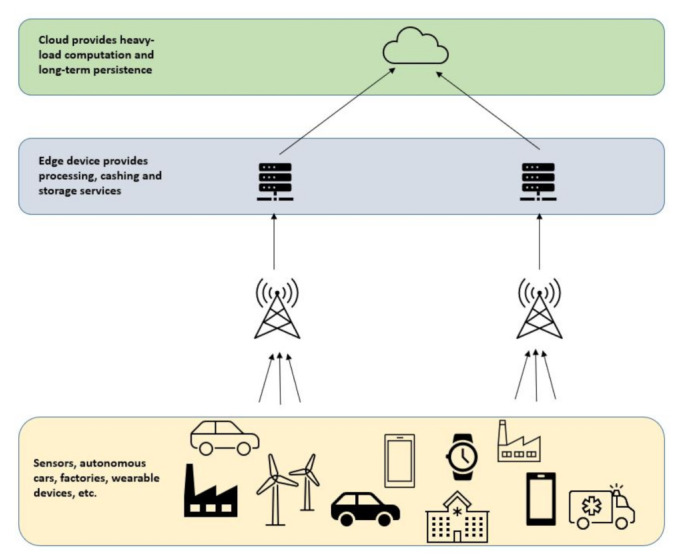
Edge computing major components.

Edge computing is premised on the idea of bringing the computation as close as possible to the data sources. Edge computing for major components in devices will provide the computation and cache capabilities that will complete that piece of the distributed intelligence puzzle. It is impossible to send all generated data to the cloud, especially if the data are ephemeral or require instant reactions. Edge devices will provide the computing and storage capabilities that are not available in the sensors layer. Such proximity to the sensors layer reduces latency and enhances real-time computation. Moreover, the great advantage of edge computing would become visible with the enhanced mobility of users or vehicles such as emergency vehicles. The basic architecture of edge computing is depicted Figure 3.

#### 3.3.3. The Role of Federated Learning

Federated learning is a variant of distributed learning. It does not replace learning methods such as deep learning or traditional ML to train and build a model. It is more of a new strategy in processing the data to build a model. FL enables different participants to train a model on their local data without sharing these data. It can break silos of valuable data, destined so far to remain segregated due to privacy regulations and laws. FL, with the other enabling technologies mentioned earlier, can realize distributed intelligent applications. However, given the fact that FL is still a nascent field, there are a number of challenges that need to be addressed to reach an acceptable level of implementation. In Section 5, Section 6 and Section 7, we provide a tutorial on FL, FL architectures in eHealth, FL challenges, and the solutions proposed so far in recent research to address these challenges.

## 4. Categories of eHealth Applications

Prior to diving deep into federated learning, in this section, we aim to explain the two prominent categories of AI-empowered eHealth applications. We find it important to describe these two categories to understand how each can adopt the suitable implementation of FL strategies.

In terms of data in the health sector, there are two major targeted data categories. First, electronic health record (EHR) data and, second, patient monitoring data or IoT-generated patient data. An electronic health record is a digital version of a patient’s paper chart or a more comprehensive report of the patient’s overall health [32]. An EHR of a patient may include the patient’s medical history such as the diagnoses of symptoms, medications, blood tests, X-rays results, immunizations, and insurance information. EHRs usually reside inside the premise of the medical entity and are stored in a structured format (e.g., database). An entity holding health data must conform to national privacy and security standards and regulations, such as the Health Insurance Portability and Accountability Act (HIPPA [33] in the US, to protect the individual’s health information.

The second major health data category is IoT data. IoT data are data generated from mobile devices such as wearable watches. These data are generated by devices carried by the user (or located in the surrounding environment) for the purpose of monitoring the user’s health. In the field of healthcare and medicine, IoT-based systems are known as the Internet of Medical Things (IoMT). IoMT envisions a network of medical devices that use wireless communication and machine-to-machine (M2M) communications to enable the exchange of healthcare data. Unlike EHRs, where data are captured upon the patient’s visit to the hospital, IoMT data are captured continuously over time. Examples of IoMT data are episodic (e.g., accelerating heart rate) or non-episodic (such as diet and weight) health signs. Both EHR and IoMT-generated data can contain Protected Health Information (PHI), also referred to as personal health information, and are subject to the user’s consent before they can be shared with a third party.

**Figure 4:**
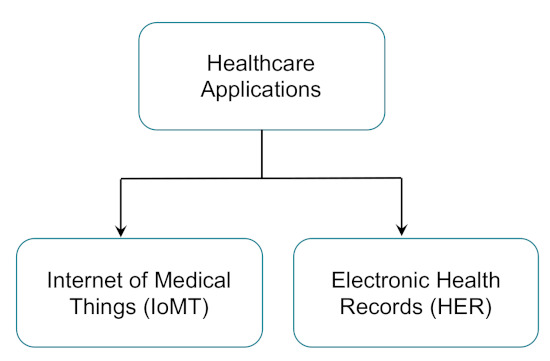
E-Health application categories.

Based on those two distinct categories of targeted health data, we categorize healthcare applications into two main categories: IoMT-based and EHR-based applications (Figure 4). In the following subsections, we explore these two different categories and point to some recent articles about applying FL in such categories. For a summarized list, check Table 4.

### 4.1. Category I—IoMT-Based Health Applications

The Covid-19 pandemic is yet another motivation for discerning the direction in which future health applications should go. Specifically, the future health applications should be designed to improve quality of life and to serve the mass of patients remotely and with minimal interventions by humans. IoMT-based systems are the embodiment of such solutions. They leverage the advancement in wireless communication, big-data analytic, and sensing technologies to make personalized healthcare services available for everybody, anytime and anywhere.

**Figure 5:**
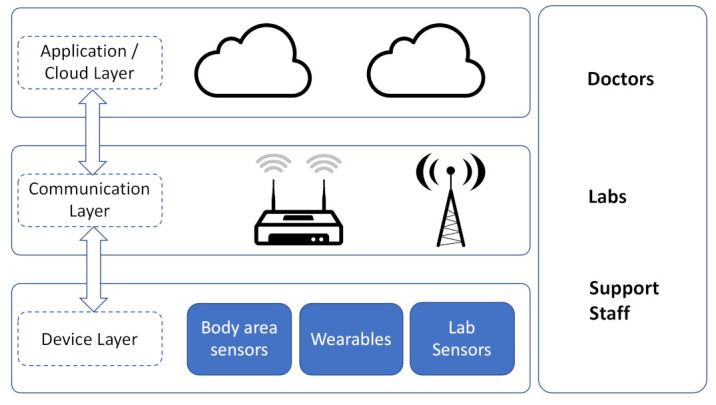
Four major parts of IoMT.

An IoMT application consists mainly of four parts, as depicted in Figure 5: a device layer, a communication layer, data layers, and users. These parts reside in a classic layered architecture, i.e., sensors reside on the device layer, which makes use of the communication bit-pipes (base station) of the communication layer to connect to the data layer (data center) and cloud resources. Ref. [34] goes into detail in describing IoMT, especially its key component—Wireless Body Area Network (WBAN). A WBAN connects the wearable sensors with the user’s mobile phone, which serves as an edge device and where some basic processing occurs. The WBAN typically expands over the whole human body and the nodes are connected via short-range wireless communications. Among different short-range communications, infrared, Wi-Fi, Ultra-wideband (UWB), ZigBee, and Infrared Data Association (IrDA), Bluetooth is currently the most suitable standard for implementation into wearable healthcare systems. Bluetooth is characterized by its low latency of 3 ms, its high data rate of 1 Mbps, its robustness against interference and noise, and its security [34].

Currently, different sensors on the market can be used with different physical indications and for different objectives, such as sensors monitoring heart rate, respiration, blood oxygen saturation, glucose, or even motion. After initial processing in the smartphone, the information gets transmitted to the cloud, where advanced processing occurs using deep learning or ML techniques. To conduct this transmission, long-range communication technology is used, namely Low Power Wide Area (LPWA) technology. LPWA emerged as a term in 2013—not as a new technology standard, but rather as a class of wireless technologies that are well suited to the specific needs of machine-to-machine (M2M) and IoT devices. The majority of IoT devices, especially those in smart city and industrial sectors, do not require the same speed and bandwidth that consumer cellular devices need. However, they do need the longevity of traditional Long-Term Evolution (LTE) cellular networks [35]. Besides the communication technology, IoMT utilizes identification technology such as RFID, QR code, or node self-identification for nodes to identify themselves prior to communication. Moreover, IoMT utilizes location technology that is very beneficial in wearable devices [36]. An endless list of applications can be developed under the umbrella of IoMT. In Section 8, we focus on examples of these applications to demonstrate the role of FL to enable them.

### 4.2. Category II—EHR-Based Applications

Medical institutions conduct deep-learning algorithms on their patients’ data to discover patterns and enhance diagnosis. For example, it can identify patterns in patient symptoms or specific types of cancer using intelligent imaging solutions. Nevertheless, there is not enough data in one organization to represent the wider population and hence extract more valuable and generalized insights. The solution is to consolidate different datasets from different medical sources and conduct deep learning on this consolidated version.

However, laws and regulations prohibit transmitting patients’ private data out of the control and hold of the owning medical entity. Here, federated learning can step in to allow integration of these data silos without the need to exchange sensitive information. Each entity would keep its dataset within the confinement of the organization, and by using FL, all entities would train the same model, then send updates of their local training to each other or to a centralized trusted aggregator. Because it is unlikely that different hospitals and medical entities would agree to have a fusion center that is trusted by every hospital to collect and aggregate their data, EHR-based applications prefer the decentralized FL architecture (also called peer-to-peer) over the distributed architecture with its central aggregator. More on this appears in Section 6.

Medical institutions will depend on federated learning in pursuit of integrating data silos from different departments or different institutions, in order to enlarge the training dataset and enhance its quality, which improves the model quality. This process is a time-bounded task that finishes as soon as the mission is completed by producing the predictive model. Accuracy of the model in these applications is highly preferred over personalization, in contrast to IoT-based applications, where personalization is of high value. The work in [37] is an example of an EHR application that developed a federated method to predict hospitalizations during a target year for patients with heart diseases, based on their medical history as described in their EHRs. The data used for the experiments came from the Boston Medical Center and consist of only EHRs. Another example of EHR application is the medical whole-brain segmentation work presented in [26].

## 5. Federated Learning Tutorial

### 5.1. An Overview

Federated learning is a machine learning variant, where multiple clients train a model collaboratively and are orchestrated by a centralized server. In this setting, the training data are decentralized. McMahan in [57] coined the term “Federated Learning” for the first time in 2016. Although federated learning initially focused on edge devices and mobile applications, the interest in applying FL to other application domains has greatly increased in the recent past. Domains include autonomous driving, eHealth, Industry 4.0, mobile operator networks, etc. Inspired by different versions of federated learning in literature, we came up with the following definition that we believe captures the crux of the idea.

**Definition** **1.** *Federated learning is a setting of machine learning allowing multiple entities (may also be termed as clients) to collaborate in solving problems, where the orchestration is carried out by a service provider or a central server. Clients do not need to send the local data to the central server; instead, a model is trained locally at clients and the hyper parameters are exchanged with the server. The process may be repeated until the desired accuracy is achieved.*

Alternatively, federated learning may be defined as:

**Definition** **2.** *Let there be N number of data owners, which is the set of distinct elements $\left\{F_{1},\cdots F_{N}\right\}$. All the elements of the set train a machine learning model on their local data $\left\{D_{1},\cdots ,D_{N}\right\}$. On the contrary to the classical (central server) method where all data is collected by the centralized server $D=D_{1}\cup \cdots \cup D_{N}$ to train a model $M_{c}$, in  federated learning the data owners collaboratively train a model $M_{f}$. In federated learning $F_{i}$ does not expose its data $D_{i}$ to other elements of the set F.*

Ideally, the accuracy of $M_{f}$ should reside in the close vicinity of $M_{c}$ i.e.,

(1) $$|M_{c}-M_{f}|<\phi$$

where ϕ is a non-negative number. Equation (1) shows that federated learning algorithm has ϕ accuracy loss.

**Figure 6:**
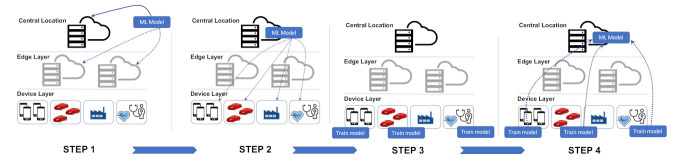
Steps of Federated Learning.

To further elaborate on the concept of federated learning, consider the four steps depicted in Figure 6, which are broken down into the following:

- **Step 1—Model Selection:** In this step, the central server selects a baseline AI model (e.g., neural network, linear regression, etc.), which we term as the global model. The global model may reside in the central server in a datacenter or in servers at the edges. The clients are then selected to train the model on their local datasets. It should be highlighted that the clients could belong to one application domain/stakeholders or multiple domains/stakeholders.
- **Step 2—Model Transmission:** In this step, the central server transmits the selected model to the clients.
- **Step 3—Local Training:** In this step, the clients that are part of the learning network train the copy of the model with client-specific data and send their learned model to the central server. This process is repeated on a periodic basis.
- **Step 4—Enriching the global model:** In this step, the central server captures and aggregates the client learned parameters and adjusted weights. Having acquired and aggregated the parameters from the clients, the central server updates the global model. The updated model is then shared again with the clients and the process is repeated until the required accuracy level is reached.

### 5.2. Types of Federated Learning

Federated learning can broadly be broken down into different categories based on (i) How data is distributed among various stakeholders in the feature and sample ID space; (ii) The involvement of stakeholders and their position; (iii) Topology type. Next, we detail these categories.

Let the matrix $D_{n}$ represents the data of client *n*. The rows of matrix represent samples, whereas the features are contained in the columns. It goes without saying that some data-sets may also contain label data. Let X denotes the feature set, Y represents the labels set, and I corresponds to the sample ID. Hence, the training data-set is (X,Y,I).

#### 5.2.1. Intra-Features and Inter-Regions Federated Learning

In this category, the datasets share the same feature set but different samples. This category focuses on applications that involve clients from different organizations, geographically distributed, and different technologies. Data are generated locally and remain decentralized. Each client stores its own data and cannot access the data of other clients. Data are not independently or identically distributed. Although the central server carries out all the organization, it just gets the hyper parameter values from the clients and has no access to raw data. Suitable communication bit-pipes are used for connectivity between the clients and a central server. Each client may participate in all rounds of the computation. Major application domains for this type of federated learning are: health, finance, autonomous driving, mobile network, etc.

**Figure 7:**
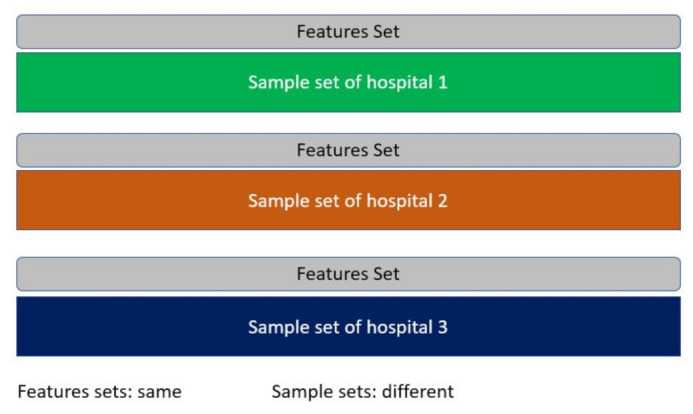
Intra-features and inter-regions FL.

For instance, let say there are a number of hospitals in a city that collaboratively want to develop a prediction model for COVID-19 or for breast cancer cases. Each hospital stores a different set of patient records, and the overlapping of samples is very low. Nevertheless, all hospitals store the records using the same attributes or features set. Federated learning in this case works with the same feature set but different sample sets. See Figure 7. Along similar lines, in an autonomous driving scenario, consider typical geographically distant road segments with road segment-specific environmental variables. Assume that the OEMs need to learn about the planning of autonomous vehicles, and road infrastructure providers wish to learn about traffic intensity in those road segments. Although the vehicles in the two segments are different, they have a features set in common.

This type of federated learning can be defined as:

$$X_{i}=X_{j},Y_{i}=Y_{j},I_{i}\neq I_{j},$$

$\forall D_{i}$, $\forall D_{j}$, i≠j

The authors in [39] used federated learning for the prediction of insurance issues, [40] looked into electronic healthcare records mining, ref. [41] looked into application of learning in pharmaceuticals discovery, ref.  [42,43] used federated learning in medical data segmentation, and the authors of [44] exploited the features of federated learning smart manufacturing. In the recent past, Google collaborated with an Android-based horizontal federated learning solution [57]. An Android user updates the parameters locally and shares them with the Android cloud. Similarly, authors in [58] proposed a deep learning scheme where the stakeholders train the model locally and share a subset of parameters with the global model. An aggregation scheme to ensure user privacy in a federated learning framework is discussed in [59]. Authors in [60] contributed with a multitask federated learning that enables multiple sites to finish separate tasks while ensuring privacy and sharing the required information for learning. A client-server structure is proposed in [57], where the federated learning allows the models to be built at client devices and collaborates at the server to build a global federated model. The contribution to improving the communication bit-pipes and the relevant costs of enabling federated learning is discussed in [61]. A similar approach relevant to communication is proposed by the authors of [62], who focused on Deep Gradient Compression to reduce the communication bandwidth requirements in large-scale distributed training.

#### 5.2.2. Inter-Features and Intra-Sample Federated Learning

**Figure 8:**
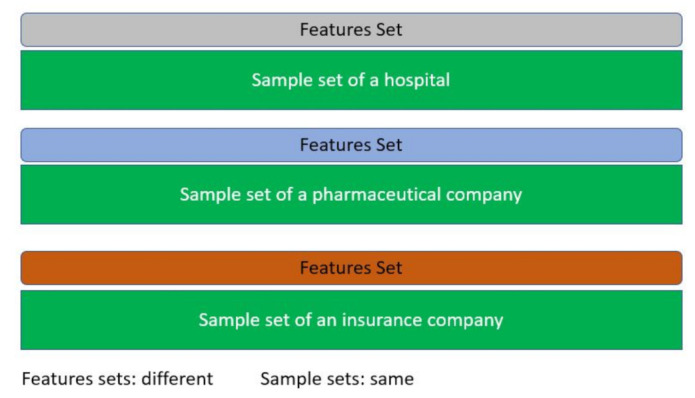
Inter-features and intra-sample FL.

In this type of learning, the datasets share similar sample ID sets but different feature sets (see Figure 8). For instance, a hospital, a pharmaceutical company, and an insurance company all want to build a prediction model that suits their business objectives based on a shared sample set of patients. The hospital wants to predict the progression of a patient case to adjust treatment, while the pharmaceutical company wants to monitor the effectiveness of their medicine on the patients on a short and long-term basis. Lastly, the insurance company wants to predict costs based on a patient’s admission and other financial elements. Each of the entities holds a different set of attributes for the same patient. In this case, FL should federate the gradients and serve each entity based on its requested goal.

$$X_{i}\neq X_{j},Y_{i}\neq Y_{j},I_{i}=I_{j},$$

$\forall D_{i}$, $\forall D_{j}$, i≠j

#### 5.2.3. Inter-Features and Inter-Region Federated Learning

This category of learning applies to the scenarios in which the datasets differ not only in samples but also in features. To understand this for the autonomous driving paradigm, consider two geographically distant road segments with different environmental variables. It is evident that the features and vehicles of the two road segments do not overlap. The transfer learning techniques turn out to be useful for the entire feature and sample space. This is to say that a common representation between the two feature spaces is learned using the limited common sample sets and later applied to obtain predictions for samples with only one-sided features. Such a type of federated learning can be expressed as:

$$X_{i}\neq X_{j},Y_{i}\neq Y_{j},I_{i}\neq I_{j},$$

$\forall D_{i}$, $\forall D_{j}$, i≠j

#### 5.2.4. Cross-Device Applications

The clients are a very large number of mobile or IoT devices. Similar to the previous type, the data are generated locally and remain decentralized. Each client stores its own data and cannot read the data of other clients. Data are not independently or identically distributed. A central orchestration server/service organizes the training but never sees the raw data. The topology used is a hub-and-spoke topology, with the hub representing a coordinating service provider (typically without data) and the spokes connecting to clients. Only a fraction of the client base is available at any one time, often with diurnal or other variations. These applications are massively parallel; with up to 1000 clients, communication is often the primary bottleneck, though it depends on the task. Generally, cross-device federated computations use wifi or slower connections. Clients cannot be indexed directly (i.e., client identifiers are not used). Cross-device apps are stateless in that each client will likely participate only once in a task, so generally a fresh sample of never-before-seen clients in each round of computation is assumed. Clients are highly unreliable; 5% or more of the clients participating in a round of computation are expected to fail or drop out (e.g., because the device becomes ineligible when battery, network, or idleness requirements are violated). There is fixed partitioning by example (horizontal).

### 5.3. Federated Averaging (FedAvg)

The traditional objective of training a dataset in machine learning is to minimize the loss function of predicting an input. Let say for a dataset containing n samples ($x_{i},y_{i}$, 1≤i≤n). Given sample input $x_{i}$, the model should predict $y_{i}$ using a set of parameters *w*. While $y_{i}$ is an estimated value, $f_{i}\left(w\right)$ measures how far this estimated value is from its true value. The less $f_{i}\left(w\right)$ is, the more confident the model is. For the whole dataset, the loss function f(w) for a model is the average of error prediction for each input. The objective is to minimize the error, hence the loss function is as presented below:

$$\min_{w}f\left(w\right)\;\;\mathrm{where}\;\;f\left(w\right)=\frac{1}{n}\sum_{i=1}^{n}f_{i}\left(w\right)$$

Stochastic Gradient Descent (SGD) is widely used to optimize this objective. Through random mini batches of the dataset for a client *k*, and iteratively, SGD keeps optimizing the objective by updating the value of *w* in small steps, as presented below:

$$w_{t+1}\leftarrow w_{t}-\eta \nabla f\left(w_{t};x_{k},y_{k}\right)$$

The new set of *w* at iteration t+1 should bring us closer to the targeted minimum value of the loss function, than what it was at iteration t. While $\nabla f\left(w_{t};x_{k},y_{k}\right)$ is the amount of movement or change at each iteration, η is the learning rate which is a tuning parameter that determines the step size at each iteration while moving toward a minimum of a loss function.

The distributed (federated) variant of SGD is called FedSGD. In FedSGD, the data is distributed across different clients, and each performs one and only one round of local computation before sending the result to the server. FedSDG is the baseline of the FedAvg. FedAvg is the standard vanilla-flavor algorithm used in the federated learning process. FedAvg differs from FedSGD in some points. First, in FedSGD a single batch gradient calculation (on a randomly selected client) is performed per round of communication, while in FedAvg clients can perform multiple gradient calculations (epochs) before sending the results to the server. Moreover, the FedSGD approach is computationally efficient but requires very large numbers of rounds of training to produce acceptable models. On the other hand, convergence in FedAvg is not guaranteed. This is because the FedAvg client does not send gradients, as is the case in FedSGD, but rather an updated model. The averaging of gradients guarantees convergence, while the averaging of the model cannot. In terms of communication, gradient averaging is a heavier consumer due to the single round per gradient Algorithm 1 presents the Federated Averaging algorithm.
**Algorithm 1** Federated Averaging. In the cluster there are *N* clients in total, each with a learning rate of η. The set containing all clients is denoted as *S*, the communication interval is denoted as *E*, and the fraction of clients is denoted as *C*
**On Server:**1:Initialization: global model $w_{0}$.2:**for** each global epoch t∈1,…,epoch **do**3:    # Determine the number of participants.4:    m←max(C·N,1)5:    # Randomly choose participants.6:    $S_{p}=RandomChoice\left(S,m\right)$7:    **for all** each client $k\in S_{p}$ **do in parallel**8:        # Get clients updated model.9:        $w_{t+1}^{k}\leftarrow OnClientUpdate\left(k,w_{t}\right)$10:    **end for**11:    # Update global model.12:    $w_{t+1}\leftarrow \sum_{k=0}^{N}p_{k}w_{t+1}^{k}$13:**end for****OnClientUpdate**$\left(k,w_{0}\right):$14:**for** each client epoch **do**15:    # Do local model training on local dataset.16:    $w_{e+1}\leftarrow w_{e}-\eta \nabla F\left(w_{e}\right)$17:**end for**18:**return**$w_{e+1}$

Notice the following:

- FedAvg gives more weight $p_{k}$ to devices with larger datasets
- Each client is required to do the same number of local epochs.

Compared to existing distributed learning schemes, FL is distinguished by several key aspects, explained in [6] as follows:

- Data are heterogeneous and must be assumed to be non-identical, independent (non-i.i.d). Since training the data on a given user is typically based on that user’s local dataset, and the local data are not representative of the population distribution, this means data are statistically heterogeneous.
- Devices are heterogeneous due to the varying computational and energy power. The heterogeneity factor has an influence on designing the learning process. Considering device heterogeneity should influence how vehicles are selected to be part of the training process and how many total local epochs each vehicle can be assigned to.
- There is no control on the participating devices, which means we can expect dropouts and unexpected behaviors of participants.
- There is a massive number of expected participants, and, due to limited communication and connectivity, FL can entail many challenges in achieving a proper convergence.

These distinguishing features of FL add many challenges to the implementation of real scenarios. We explore these challenges in Section 7.

## 6. FL Architectures in eHealth

**Figure 9:**
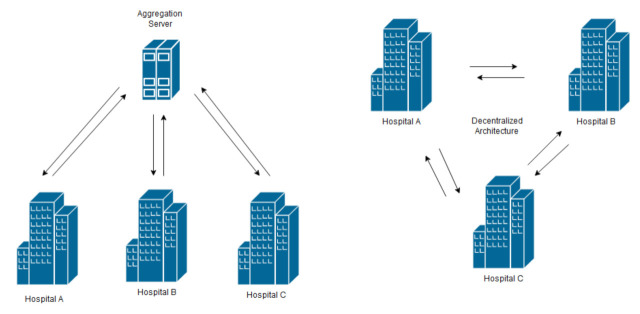
Distributed FL vs Decentralized FL.

There are two commonly adopted FL architectures in healthcare applications: (1) the conventional distributed and (2) the decentralized architecture [24]. See Figure 9.

The traditional FL architecture is distributed by a star network that carries a centralization feature embodied in the aggregation actor. In distributed architecture, the participants independently train the model and send the updates to an aggregation server. The aggregation role is conducted by an external, trusted third-party or server. The aggregation server is responsible for collecting the updates from each of the participants, combining them, and producing a new version of the model. On the other hand, in decentralized architecture, also known as peer-to-peer FL approach, there is no single point where the decision is made. The strategy of keeping this role within an external server or delegating it to the participants is mainly dependent on the feasibility of full agreement of all parties (e.g., hospitals) on one trusted, external third-party, or for one of them to play the aggregation actor. Another factor is the type of data being processed—whether it is EHR or IoT-based data. Therefore, we find that most of the proposed solutions in research that deal with EHR from different hospitals adopt the decentralized, not the distributed, approach.

For example, in [38] the researchers opted for decentralized rather than distributed for their EHR-based model. They justified this option due to the fact that in a set of different hospitals it would not be feasible to have a fusion center that is trusted by every node (hospital) to collect healthcare data. Instead of using the traditional SGD optimization algorithm with one master and many slaves, they applied fully decentralized, nonconvex stochastic algorithms for federated learning and obtained reasonably good results for health record datasets. In terms of topology, instead of using the typical FL star network, they used a well-connected graph network. Ref. [45] also built its solution using the decentralized approach in working on medical images that resided in different sources. The proposition acknowledges that the variation of medical images, combined with the limited number of medical images, would cause significant variation of parameters updated by clients, leading to bad convergence after aggregation. The core idea of their solution was to alleviate the variations among different clients by transforming the raw medical image data of all clients onto a common image space, via image-to-image translation, without violating the privacy setting in FL.

In terms of performance, of the two, decentralized could be the better solution as we find in [26]. In this paper, the authors introduced BrainTorrent, an FL framework without a central server. It trained a complex, fully convolutional neural network in a decentralized fashion using whole-brain image segmentation with 20 classes having severe class imbalance. Through experiments, they proved that BrainTorrent achieved a better performance than FL with servers under different experimental settings. The typical network topology in decentralized FL architecture is a fully meshed network. Nevertheless, different topologies have been used and some proved out-performance. For example, ref. [63] proved, through empirical results and a theoretical investigation, that the Erdos-Renyi graph, if used as the network topology for a decentralized FL architecture, can outperform a fully meshed network. They concluded that the network topology used in decentralized FL has an impact on the performance of the model convergence as they conducted their comparison with other prevalent networks, namely Scale-Free Networks, Small-World Networks, and Fully Connected Networks.

Although decentralized FL is preferred in EHR settings, it is applicable for IoMT-based data settings, as we find in [64]. The authors here proposed a decentralized Framework for Human-Computer Interaction for IoMT Applications. Some other papers, which also adopted a decentralized federated learning design, focused on enhancing the model convergence. For example, ref. [65] proposed utilizing a segmented gossip approach, which not only makes full utilization of node-to-node bandwidth but also has good training convergence by carefully forming dynamical synchronization gossiping groups. On the same concept of decentralization, ref. [66] utilizes blockchain technology for model training. Their architecture suggests exchanging and verifying local learning model updates between participants. In their model, you can think of the typical FL training rounds as Blockchain Blocks and the model updates as transactions in blocks. The aggregation, in this case, is not taking place in an external unit or server—rather, each participant aggregates the model using the updates in the previous or latest blocks. Decentralized blockchains are immutable, which means that the data entered is irreversible. This system also inherently makes an irreversible timeline of data when implemented in a decentralized nature. When a block is filled, it is ingrained as part of this timeline. Each block in the chain is given an exact timestamp when it is added to the chain [67].

For the other team, the “distributed strategy” team, the drive behind keeping the aggregator role independent depends on different factors, but one major one is the nature of the data. In the “decentralized case”, EHR is of a static nature, meaning it does not change every second or every day. Records reside in the database of the hospitals and the process of training between different hospitals can rely on a good communication and connection channel. Moreover, the accuracy of the model is of higher priority than personalization. On the other hand, the data in the “distributed approach” is mainly collected from scattered distributed users or devices. The number of participants is way greater than that in the “decentralized” case. Moreover, data is representing individuals’ personal activities, and the model is expected to respond to these activities with a high level of personalization. That is why we find the solutions that deal with IoMT or mobile devices such as wearable watches resorting to the distributed FL approach instead. Concrete examples about applying distributed FL in wearable devices can be found in Section 8.2.

## 7. Challenges of Federated Learning and Relevant Research Works

Although FL addresses the crucial requirement of preserving privacy, a number of challenges still have to be addressed for FL to achieve its full potential. The following is a list of major challenges.

### 7.1. Heterogeneous Characteristics of Clients

The central servers in the classical machine learning approaches are equipped with rich computation and storage infrastructure. Clients may have differing characteristics (e.g., storage, computation power, and communication capabilities including interfaces, support for throughput, battery life, etc.) of edge and device layer entities including smart phones, vehicles, body area sensors, industrial sensors, etc. This becomes a challenge in federated learning, as this may result in varying learning time and resource availability. Application domains with a large number of clients usually suffer from such heterogeneously equipped device layers, resulting in asynchronous updates toward the server. This introduces delays in aggregation at the server.

### 7.2. Communication Cost

Although in federated learning there is no need to communicate raw data with a central server, and hence there is less overhead on the network, there is still a need to transmit millions of parameters between the participants and the server. This poses an overhead on the communication channel, especially if we consider the multiple communication rounds needed to achieve model convergence. Moreover, unreliable network connectivity and limited bandwidth can add to the communication cost. In addition, complementing FL with other privacy-preserving techniques such as differential privacy or encryption adds to the size of the updates and hence to the communication costs. The FL training process encompasses different steps, and each has an impact on communication. Any of the following can cause either an increase or decrease in the number of communication rounds or in the size of the model (i.e., parameters exchanged):

- The design of the deep network model being trained (e.g., number of layers)
- The participants’ selection—their data quality, their computation power, and the number of participants at each round
- The decided local epochs for each vehicle
- The frequency of sending updates

Based on the above, strategies adopted by research so far to reduce communication cost in FL are focused on:

- reducing the total number of communication rounds, or
- reducing the size of transmitted messages at each round.

**Figure 10:**
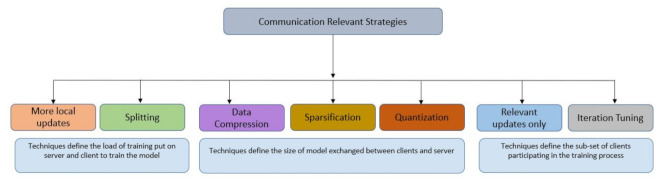
Solution approaches for communication relevant challenges.

Inspired by the trends in the research literature, we suggest three major solution categories, namely: (i) Techniques that define the load of training put on the servers and the clients to train the model (ii) Techniques that define the size of the model exchanged between the clients and the servers (iii) Techniques that define the sub-set of clients participating in the training process. Figure 10 depicts these solution dimensions.

The first technique assigns more local updates on a device before sending for aggregation [68,69]. On this same subject, another strategy focuses on finding a balance between global and local training. The strategy is based on partitioning the deep learning process into parts and outsourcing some of the parts to a more computationally powerful server, either in the edge or in the cloud such as in [70,71,72,73].

In regards the data compression techniques, quantization and sparsification, the aim of these techniques is to reduce the size of the updates that are exchanged between clients and servers. Research works [74,75,76,77,78,79,80,81,82,83,84,85], are some of the major approaches contributed in this regard. For the “fewer rounds” and ”relevant updates only”, algorithms are developed to keep a balance between the number of communication rounds and their relevancy, and the model accuracy. Research works in this area include [38,86,87].

### 7.3. Varying Client Sets

The assumption that all the clients remain active all the time may not hold in many cases. For instance, in autonomous driving settings, the clients, i.e., autonomous vehicles, in specific regions may not be part of FL for every iteration. Hence, a more realistic assumption is that only a fraction of the clients will be part of the learning at a particular time. Furthermore, those active clients may also drop out of the learning process due to poor connectivity or inadequate computing resources. Major problems under this category are accurately estimating active clients and creating a framework supporting hardware with heterogeneous characteristics.

### 7.4. Statistical Heterogeneity

With varying data from the involved clients, the distribution is usually non-identical. This is because the clients capture or generate data in a non-identically distributed fashion across the domain. For example, in eHealth settings, the data captured via wearable devices vary from individual to individual. Furthermore, the number of data points for different devices may significantly vary, which negates the independent and identically distributed (I.I.D.) assumptions usually used in distributed optimization. It also increases the probability of stragglers and complexity. Data heterogeneity can cause non-trivial performance degradation in FL, including up to 9.2% accuracy drop, and 2.32× lengthened training time [88]. Beside divergence, heterogeneity can cause unfairness in accuracy across devices, and can produce an un-personalized model. Research works that focused addressing this challenge include [87,89,90].

### 7.5. Privacy Concerns

It is no secret that the healthcare industry is one of the most targeted industries for privacy and security attacks. The return of these attacks for adversaries is very profitable, with medical records worth between 10 to 20 times the value of credit card data [91]. Authors in [92] interestingly argue the major incentives that motivate prospective bad actors to carry out these attacks in the medical deep learning systems. Referring to the US health system, they basically prove that the entities of the medical ecosystem are shifting toward total dependence on Deep Learning (DL) algorithms to make decisions, whether administrative or medical. Specifically, one of the key players, the insurance companies, would rely on DL/FL systems to decide on reimbursement requests, which are worth billions of dollars in the US health economy. Understandably, many would like to have an influence on the outputs of these systems for their own benefit.

Because of the inevitable shift toward more investment in deep learning systems in the health sector and possible wider adoption of FL, the scope of this section will focus on the techniques for countering adversarial attacks on deep/federated learning and on countering techniques during and after the training of the model. Privacy and security mechanisms, including authentication, authorization, and access control, are other techniques that are also used to preserve the privacy of sensitive healthcare data, but they are out of the scope of this paper. For more on these techniques and others applied in IoMT systems, we advise reading surveys [93,94] In this section, we explain the potential attacks on DL/FL health systems and review and compare the countering strategies.

Federated Learning (FL) enables multiple participants to train a machine learning model collectively without directly exchanging the data. Although FL appears to ensure that data remain on-premises, recent studies have shown that there is still the possibility of an actor exploiting the shared updates to extract confidential data, maliciously influencing the model output, or causing other harm such as model malfunctioning. Based on the timing of the attack in respect to the model life cycle, major attacks can be categorized into:

- Attacks taking place during the model aggregation phase [19,95,96]
- Attacks taking place after the model is deployed [97,98,99,100,101,102]

### 7.6. Data Labeling

Data labeling and pre-processing are important stages of machine learning. For instance, the supervised learning models demand that data are clearly labeled. This is obviously challenging to achieve across various clients of federated learning. Hence, it is imperative to design and develop model data pipelines that apply labels in a standardized way, based on events and user actions.

### 7.7. Model Convergence Time

The convergence time of federated learning is typically longer than that of the locally trained models. The factor of unpredictability fueled by the challenges mentioned above includes unreliable connection, heterogeneous devices, varying software versions, varying applications, etc.; these all add to the complexity and, consequently, to the convergence time. For this reason, federated learning solutions are typically most useful when they provide meaningful advantages over centrally training a model, such as in instances where datasets are extremely large and distributed. Some of the research works focused on this challenge [103,104,105,106,107,108,109].

### 7.8. Personalization

The QoE is highly associated with how a service accommodates the unique needs of the user. Personalization is the key to a better user experience. In FL, where all users will receive the same global model, some personalization can be lost in the middle. Therefore, finding solutions to address the personalization matter is important. Research works include [110,111].

### 7.9. Variants of FedAvg to Address Challenges

The solutions proposed in research works, to address the above mentioned challenges, vary in approach, but mainly target different aspects of the vanilla-version FL averaging algorithm, FedAvg. We opted to breakdown the targeted aspects of FedAvg into:

1. The participants’ selection algorithm
2. The model broadcast
3. The on-client update algorithm
4. The on-server aggregation algorithm
5. The synchronization schemes.

In Table 5, we list the research works and the targeted aspect in the FedAvg.

## 8. Selected IoMT-Based Health Applications

In this section, we cherry-pick few examples of IoMT-based health applications to demonstrate the design requirements and the integration of 6G, Edge computing, and FL needed to enable them.

### 8.1. Tele-Surgery or Autonomous Surgery

“Democratization of skillful surgeons” could be the best term to describe telesurgery, especially with the increased population and the shortage of skilled surgeons. Telesurgery basically allows surgeons to operate on patients who are distantly located via a robotic onsite surgeon. This technology will increase patient access to highly skilled surgeons by eliminating geographical barriers and saving time and cost. Such technology cannot be more wanted and relevant than it is today with the current situation of COVID-19, where people’s mobility is restricted, air flights are banned, and limited interaction is advised. Imagine what telesurgery can offer in such scenarios. For example, telesurgery can enable three geographically separated surgeons to operate on the same patient, who might also be in a fourth location.

A virtual operating theater can bring the surgeons, the patient, and the medical staff all together. The virtual room would display the patient’s vital signs on its virtual walls, as well as other critical physiological data. In the middle, a holographic image of the organ or field that’s undergoing the surgery would be reflected for all. Such technology would enable surgeons to communicate and perform with more accuracy and precision due to the comprehensive and high-fidelity display of the holograms. In the real operation room, where the patient is located, a robotic surgeon acts as per the surgeons’ commands. These commands would be triggered using gloves worn by the surgeons. A glove would translate the motions of a surgeon’s fingers into commands for the robotic surgeon to follow, and the glove would also transmit real-time sensing for the surgeons. This feedback, transmitted by the gloves back to the surgeons, would enable them to actually sense the feel of tissues, blood, and organs.

**Figure 11:**
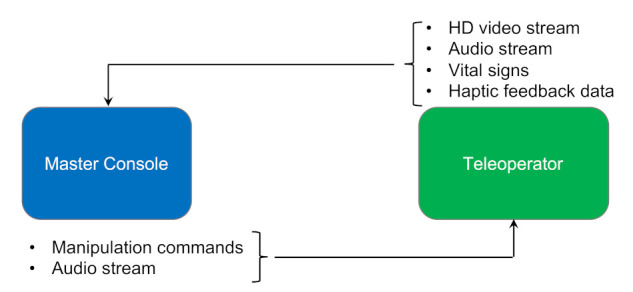
Feedback loop in telesurgery.

Ref. [46] explains telesurgery as depicted in Figure 11. A tele surgical system will consist of a master console and a slave robot, i.e., a teleoperator. A master console is an interface that is used by the real surgeon and is composed of a haptic device for position-orientation input, a video display and headphones for video and voice feedback and in the future, it will have haptic feedback output. On the other end point, the salve robot, the teleoperator is equipped with a 3D video camera and a microphone and will have several force sensors and tactile sensors available in the future.

So, telesurgery will rely on two major technologies:

- visual display (3D high-definition video or holographic objects) of the surgical field
- haptic feedback technology to translate the real surgeon’s control movements into commands to operate the on-site robotic surgeon.

Holography provides better communication for surgeons due to the extensive visualization of a patient’s body using high resolution. It helps surgeons to examine different body parts like the liver, brain, skeleton, heart, lungs, nerves, vascular system, and muscles. Holography can digitally store patient data and provide a massive amount of detailed information [51]. On the other hand, haptic devices are used to send and receive real-time physical sensation data. This helps doctors to feel real-time sensations remotely. They convert the human input to the tactile input using tactile coding [49]. Another promising technology to be added to the two for even a better experience is Augmented Reality and Virtual Reality AR/VR. AR provides a customized depth that can enable the doctor to visually zoom inside the displayed object, e.g., liver, heart, or bone. On the other hand, VR helps in creating one’s own artificial environment, as we suggested in the scenario of the three geographically separated surgeons. Because telesurgery will deal with different data formats such as video, audio, physiological data, or haptic feedback, each has different requirements in terms of latency, data rate, and packet loss. As per [47], less than 200 ms end-to-end latency is acceptable for non-haptic telesurgery. This end-to-end latency requirement gets dramatically less for haptic-telesurgery. As per [48], the minimum latency requirements for haptic data, 3D video, and vital signs would be as presented in Table 6.

As per [47], latency should be even more reduced with less than 1 ms latency and with improved reliability of 99.999 percent. We summarize the non-negotiable communication requirements for telesurgery as follows:

We summarize the non-negotiable communication requirements for telesurgery as follows:

- Ultra-low latency: because telesurgery is beneficial over long distances, the greatest challenge to implement it is the latency. Achieving less than 1 ms round-trip latency over long distances under 5G is nearly impossible, therefore a better performance is expected from 6G with its extremely reliable and low latency communication (ERLLC).
- Ultra-high bandwidth: a minimum of 1 Tbps is required to stream the ultra-high-definition images and videos [30].
- High-precision stream bundle synchronization: the network needs to manage a massive number of synchronized streams originating from different sensors, an object at different angles, or a processed volumetric fusion [50].
- Ultra-high reliability: reliability is related to the capability of a network to carry out a preferred operation with very low error rates.

The current state-of-the-art healthcare system is unable to provide telesurgery due mainly to communication issues. 6G can provide these capabilities via its core services of Enhanced Mobile Broadband (eMBB), Extremely Reliable and Low Latency Communication (ERLLC), and Mobile Broad Bandwidth and Low Latency (MBBLL) [30].

Federated learning can enrich telesurgery with pre-trained models to enhance the efficiency of the operations in the operation room, and also to reduce the human errors with predictive and prescriptive models. Think of a model that is trained using the medical history of the patients with similar health condition across different health institutions. Or another model that is pre-trained using previous surgeons’ decisions made in a similar surgery across different health institutions. Such models can assist the surgeons prior and during the telesurgury. The former, can give the surgeons a 360 view of the patient’s health status which can help detecting overlooked critical points that the surgeon might not have looked at. While the latter, can assist the surgeons in making the highly beneficial decisions when they are in doubts.

### 8.2. Wearable and Implantable Devices

Wearable or implantable devices are other examples of IoMT. They allow patients to send their health information to doctors to better diagnose diseases and track and prevent chronic illnesses. Proactive monitoring helps in predicting the likelihood of expected disease development, such as predicting a heart attack, or an epilepsy fit before it happens. Examples are:

- An insulin pump.
- A wearable smart asthma monitoring device.
- A watch to monitor depression.
- A blood pressure monitoring device.
- An ECG monitoring system.

For a comprehensive survey and classification of commercially available wearables and research prototypes we advise reading [52].

The current devices on the market are still limited in fulfilling the envisioned objective, mainly due to the wireless communication limitation. Therefore, current research is focused on enhancing the sensing techniques to become fully automatic, more accurate, reliable, cost-effective, and noninvasive. These devices gather data from very different appliances, from nano to macro, and communicate in heterogeneous types of channels, from chemical to radio or free-space optical [53].

Design requirements of such solutions would be:

- Ultra-low-power communication: unlike telesurgery, where surgery time is bounded and hence higher power consumption communication is expected and can be accommodated, in wearable or, especially, implantable devices, the longevity of battery in the sensors and other devices is very important. Part of the user experience and convenience is to limit the need for replacement or recharging of these nodes. We see improvement in battery life, but this is not enough. For continuous and smooth remote monitoring, energy-efficient schemes such as in the algorithm or protocol for communication are required [54]. For example, in [119], a prototype is introduced for a wrist-worn device for the monitoring of pervasive personalized environmental parameters. For the objective of extending the battery life, when the level of battery power is decreased, the sampling rates of gas sensors and sound level detectors are reduced accordingly.
- Highly accurate localization and positioning. Such a high level of accuracy can be achieved through THz technology with simultaneous localization and mapping-based techniques. Compared to 5G meter-level positioning precision, 6G will provide 10 cm indoor and 1 m outdoor precision in positioning [28].
- Interoperability: or the ability of heterogeneous devices to communicate and exchange data. Lack of universal standards for interoperability in the different levels (e.g., application, network) can impede IoT success [29]. Nevertheless, some effort has begun by standardization bodies such as OMA [120], 3GPP [121], and ETSI [122].

Compared to the EHRs, which represent a limited volume of data that reside in silos in each health institution, the data from wearable devices represent a much bigger, diverse, and actual today’s real-world patient population. However, what remains common for both is that data privacy must remain intact. Here comes the role of FL. FL will enable training a model collaboratively by thousands or even hundred of thousands of wearable devices in an efficient and economical way. Achieving robust implementations of FL in this scenario can make a huge progress in the massive and remote healthcare system. A robust and matured FL implementation will not only preserve the privacy but also achieve a high accurate model fast and personalize the experience of each user. The ambition is high, and hinges on overcoming the challenges of FL detailed in Section 7. In this use case, both model accuracy and personalization is of high importance especially if the application is intended to monitor a critical input. This will require a constant efforts to enhance the model as the dynamics of the environment changes. The efforts will be between the AI/ML model developers and the medical experts, to decide on the ever-changing vital and environmental relevant data from diverse groups of patients. We witness more advances in the compute power of the wearable devices and also the sensors, which will contribution to expedited FL adoption in eHealth. In the research works along this goal, we find authors in [55], proposing FedHome, a novel cloud-edge based federated learning framework for in-home health monitoring. It learns a shared global model in the cloud, which serves as our centralized aggregator, from multiple homes at the network edges, and it achieves data privacy protection by keeping user data locally. The work in [56] also utilizes the distributed approach by proposing a federated transfer learning framework for wearable healthcare, named FedHealth. First, the cloud model on the server is trained based on public datasets. Then, the cloud model is distributed to all users, where each of them can train their own model on their data. Subsequently, the user model can be uploaded to the cloud to help train a new cloud model by model aggregation. Finally, each user can train personalized models by utilizing the cloud model and local data. They use transfer learning to deliver a personalized model for each user.

### 8.3. Intelligent Healthcare Empowered by Autonomous Vehicles

By intelligent healthcare empowered by autonomous vehicles we mean a fleet of autonomous vehicles, where each vehicle is equipped with medical equipment, sensors, and actuators to transmit what is happening inside the vehicle to remote medical teams. From a centralized management platform, the healthcare provider will orchestrate dispatching the vehicles and the assignment of the relevant medical teams. Not all vehicles will be equally equipped. Some will have more sophisticated sensors and equipment as per specific health cases. Therefor, the dispatching system will be smart enough to predict where the service is highly needed and which vehicle to dispatch. The autonomous vehicle will drive to where the patient is and the medical team will commence the treatment immediately. In a case for the need to drive the patient for the hospital, then the vehicle will do this, serving as an autonomous ambulance. The solution requires extremely reliable and low-latency communication to broadcast in real time the monitored sensitive data. Such use case is complex and entails achieving high resolution perceptions for what is happening inside the vehicle and outside the vehicle. The external perception, is mainly contributing to achieve level 5 autonomous driving, whereas the internal perception is to visually communicate to the medical team the monitored patient’s status. In this paper, we focus on the role of FL to achieve autonomous driving, as an instrumental part to achieve innovative mass healthcare provision.

**Figure 12:**
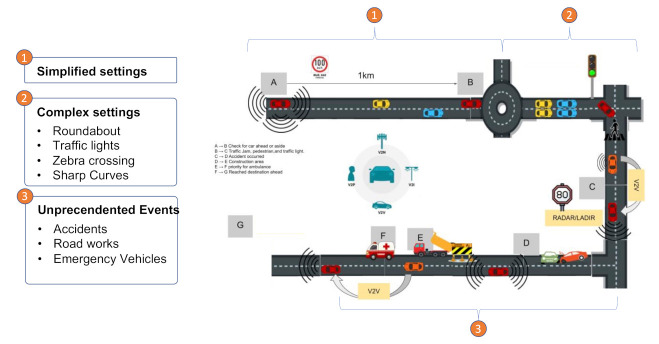
Dynamics of the complex environment, to be addressed by the level 5 autonomously driven vehicles.

The idea of future mobility and autonomous driving rests on the capabilities of vehicles to understand their environment and to react to the dynamic events of the environment, which we term as the vehicle’s perception or situational awareness. Furthermore, the classic mobility paradigm will shift to the autonomous and connected mobility paradigm, i.e., autonomous vehicles and multi-modal mobility sources under an umbrella mobility solution that also caters to intra-supermarket spaces, intra-hospital spaces, intra-airport spaces, etc. Such envisioned mobility approaches demand innovative solutions on multiple fronts, including achievement of the objectives of level 5 autonomous driving, dynamically adaptable and autonomously handled inter-stakeholder relationships, and intelligently orchestrated ITS services, etc. Hence, enhancing existing solutions and developing innovative solutions for autonomous driving and smart mobility are the two major fronts. The operations of the perception, planning, and control layers of autonomous driving that target the level 3 or level 4 autonomous driving capabilities need to be evolved to achieve the capabilities of fully autonomous driving. To highlight the challenges of level 5 autonomous driving, consider Figure 12. The figure breaks down dynamics into different road segments. Road segment A → B contains simplistic dynamics i.e., autonomous vehicle may need to execute critical maneuvers like barking, lane changing, etc. Road segment B → C represent more complex dynamics that are challenging to address for autonomous driving. These dynamics include: road congestion, traffic light, sharp turns, pedestrian passage just after the sharp turn. Road segments C → D and D → E depict the dynamics of unprecedented events e.g., accident, emergency vehicles, etc.

In what follows about, we sketch the design goals for the envisioned services of smart mobility and autonomous driving, focusing on enhanced perception. With the information and sensory data from on-vehicle sensors, the vehicles are able to create a perception of their environment. Vehicles’ perception together with the capability of interacting with other vehicles does allow some level of automation but relies on the vehicle’s visibility. It is unclear as to how capable autonomous vehicles can cope with different situations and environments. Autonomous driving does pledge an increase in road safety. The fact that a vehicle’s sensors are a limiting factor on the quality and extent of the vehicle’s perception is, however, detrimental to that pledge of increased safety. State-of-the-art approaches for vehicle perception may not suffice for the dynamics of complex environments. Furthermore, the perception for specialized use-cases like platooning, remote driving, etc. is different and needs additional sources of information and infrastructure.

Ideally, the perception creation and understanding of the environment should be very accurate and in real-time in all the complex scenarios. Such design goals may be achieved by extending the vision and enriching the understanding of autonomous vehicles through (i) external creation of a perception of the road segments/spaces through online deployed sensors and other information sources, (ii) crowd integrated perception creation, (iii) improved sensory data fusion techniques, (iv) evolving…The challenging question is: Will autonomous vehicles be able to cope with unprecedented and complex situations—roads with unregulated traffic, temporary or dynamic obstacles, vulnerable road users, sharp turns, etc.? Figure 12 captures these dynamics by defining different road segments, e.g., segment 1 is a simplified setting (a straight road with clear road markings). Autonomous vehicles operate on knowledge from past experiences, either built in by engineers or through training using machine learning. However, not all variables and situations for decision-making are known in advance. Relying on the information from on-vehicle sensors alone or implementing pre-trained reactions to events may not suffice to achieve the goals of level 5 autonomous driving [123].

The role of FL to achieve level 5 autonomous driving is promising due to different capabilities. First, its ability to train models while preserving privacy, which is important to achieve high resolution perception that involves people’s faces or sensitive information. Second, FL can efficiently reduce transmission overhead in such highly sensitive real-time application due to the fact it transmit parameters not data. Moreover, FL has potential to build models that adapt quickly to the ever-changing road conditions, leveraging the massive inputs from different sensors and edge devices scattered along the road.

## 9. Conclusion and Future Discussion

The eHealth applications of the future will be characterized by being real-time, intelligently distributed, and privacy preserving. To achieve these features, we shortlisted three major enabling technologies that need to interplay to achieve the goal. These technologies are 6G communications, edge computing, and federated learning. In this paper we focused on exploring the role of federated learning in realizing future eHealth applications. Federated learning is promising to lift some current gaps and restrictions in data privacy regulations and data silos. However, for a full adoption of FL in eHealth, many challenges need to be fully addressed. We explained these challenges and the research efforts conducted so far to address them. For healthcare providers to adopt FL, a learning strategy need to be developed. It can start between a set of different health institutions to develop a specific model. A mass adoption can follow later with partnered use cases that involve different stakeholders such as healthcare providers, governments, and communications service providers.

## Figures and Tables

**Table 1:** Abbreviations and Acronyms.

Abbr.	Full Text
AL	Active Learning
AR	Augmented Reality
DAI	Distributed Artificial Intelligence
DP	Differential Privacy
DRL	Deep Reinforcement Learning
E2E	End-to-End
EHR	Electronic Health Records
ELPC	extremely low-power communications
eMBB	Enhanced Mobile Broadband
ERLLC	extremely reliable and low latency communication
FeMBB	further-enhanced mobile broadband
FL	Federated Learning
HE	Homomorphic Encryption
HIPPA	Health Insurance Portability and Accountability Act
IoMT	Internet of Medical Things
IoT	Internet of Things
ITS	intelligent transportation system
LDHMC	long distance and high-mobility communications
LPWA	Low Power Wide Area
M2M	Machine-to-Machine
MBBLL	mobile broad bandwidth and low latency
mBBMT	massive broad bandwidth machine type
MEC	Mobile Edge Computing
mLLMT	massive low latency machine type
QoL	Quality of Life
QoS	Quality of Service
SMPC	Secure Multi-Party Computation
THz	Terahertz
uHDD	Ultra-High Data Density
uHSLLC	Ultra-High-Speed with Low-Latency Communications
uMUB	Ubiquitous Mobile Ultra-Broadband
URLLC	Ultra-Reliable Low-Latency Communications
VR	Virtual Reality
WPAN	Wireless Body Area Network

**Table 2:** Comparative Summary of recent FL Surveys.

Ref	Federated Learning						
	Tutorial		Challenges and Solution Appraoches			Technological Discussion	Application Domain(s)
	High Level	Detailed	Device Heterogeneity	Data Heterogeneity	Communication Relevant		
[11]		X			X	Partially	Agnostic
[12]	X				X	Partially	Agnostic
[13]	X			X	X		Agnostic
[14]	X		X	X	X		Agnostic
[15]		X	X	X	X		eHealth partially
[16]		X					Agnostic
[14]	X				X	X	Agnostic
[19]	X						Agnostic
[20]	X				X	Partially	Vehicular IoT
[23]		X					Healthcare Informatics
[24]	X			X			Healthcare Informatics
Our Paper		X	X	X	X	X	Healthcare Informatics and IoMT

**Table 3:** Reflects the latency, data rate and reliability of telesurgery enabling technologies.

Capability	5G	6G
Per device peak data rate	10 Gbps	1 Tbps
E2E Latency	10 ms	1 ms
Mobility Support	Up to 500 km/hr	Up to 1000 km/hr
Satellite integration	No	Fully
THz	Very limited	widely
Energy efficiency	1000X relative to 4G	>10X relative to 5G
Reliability	99.9%	>99.999%
Traffic density	10 TB/s/km^2^	>100 TB/s/km^2^
Positioning precision	Meter level	Centimeter level

**Table 4:** Articles about FL applications in Healthcare domain.

Application	Ref.	Focus
EHR-based (e.g., medical images) prediction models	[37]	EHR application to predict hospitalizations for patients with heart diseases
	[26]	EHR application for medical whole-brain segmentation
	[38]	EHR-based model
	[39]	federated learning for prediction of insurances
	[40]	electronic healthcare records mining
	[41]	application of learning in pharmaceuticals discovery
	[42,43]	use federated learning in medical data segmentation
	[44]	utilizes the features of federated learning in smart manufacturing and healthcare
	[45]	decentralized FL approach working on medical images
	[26]	BrainTorrent: a FL framework to train a complex fully CNN in a decentralized fashion using whole-brain image segmentation
Telesurgery and its design requirements	[46]	Tactile Robotic for Telesurgery
	[47]	Telesurgery and its enabling technologies
	[48]	Communication requirements for telesegury
	[49]	Tactile-based Telesurgery
	[30]	Communication requirements for telesegury
	[50]	Holography for telesurgery
	[51]	Holography applications toward medical field
IoMT and wearable devices applications and their design requirements	[52]	survey of commercially available wearables
	[53]	a survey about IoMT
	[54]	ECG monitoring systems
	[28]	communication requirements for IoMT to be provided by 6G
	[29]	communication requirements for IoMT provided by 5G
	[55]	FedHome: a cloud-edge based federated learning framework for in-home health monitoring.
	[56]	FedHealth: federated transfer learning framework for wearable healthcare.

**Table 5:** Variants of FedAvg in the recent research work and their modification focus.

	Clients Selection	Model Broadcast	On-Client Update	On-Server Aggregation	Synchronization Scheme	Appraoch
**Data Heterogeneity**						
FedProx [103]			X			add a proximal term to the client cost functions, thereby limiting the impact of local updates by keeping them close to the global model.
SCAFFOLD [104]		X				the server broadcasts the model to the clients along with its control variate c.
			X			each client calculated the difference (c- ci) which is an estimate of the client-drift and is used to correct the local update.
q-FedAvg [105]			X			propose a new objective for the local loss function that penalizes the devices with poor performance to achieve better fairness,
Per-FedAvg [110]			X			show how the fundamental idea behind the Model-Agnostic Meta-Learning (MAML) framework can be exploited to design a personalized variant of the FL problem. Changes the local loss function of a client
PeFedMe [112]			X			using Moreau envelopes as clients’ regularized loss functions, which help decouple personalized model optimization from the global model learning in a bi-level problem stylized for personalized FL
IFCA [106]			X			the client estimates the cluster membership from the models sent.
				X		the server aggregates the received models per cluster
FedMax [89]	X					introduce a similarity-based worker selection approach, which chooses the most effective workers with least dataset similarity (e.g., more non-IID). As data is not accessible by the central server in federated learning, FedMax smartly implies dataset similarity from workers’ updates.
					X	realize a relaxed synchronization communication scheme with a workload balancing mechanism by taking heterogeneous computation capacities of workers into account.
FAVOR [87]	X					dynamic device selection using DRL
VKN [90]	X					an orchestration method to intelligently select the proper vehicles required to train a model. The heart of their proposed orchestration method is a Vehicular Knowledge Networking framework
[86]					X	sending only relevant updates for aggregation
[113]					X	only “fine” local models are sent for aggregation
[114]	X					To further reduce communication, they group the CSs into clusters before applying the FL algorithm
**Communication**						
FedPaQ [84]			X		X	combine both periodic averaging and model compressing using quantization
FADL [70]			X			train the first layers of the neural network model using data from all sources in a federated learning manner and the other parts locally using local data from specific data sources
Split Learning [71]			X	X		split the layers of the network between the client and the server, where the server does the heavy load
PRCL [72]		X				split the model into three parts and outsourcing the computationally heavy part to the cloud and using homomorphic encryption
FetchSGD [74]		X	X	X		use Count Sketch to compress model
FedPaQ [84]			X	X	X	combine both periodic averaging and model compressing using quantization
[38]			X			perform local updates for several iterations and then enables nodes to communicate with each other
PFL-IU [86]				X		remove irrelevant updates instead of compressing them
[115]	X					Active Learning for devices selection
[109]			X			Active learning to speed up model convergence
**Privacy and Security**						
[19]				X		identify malicious participants via their model updates
[98]			X			use differential privacy to perturb the gradients as a defense layer against reverse engineering
[99]			X			mitigate the noise introduced by differential privacy
[116]		X	X	X		adopt Multi-Party Computation (MPC) to achieve privacy-preserving model aggregation for FL
[55,56,72,117,118]		X	X			adopt homomorphic encryption

**Table 6:** Minimal communication requirements for telesurgery.

Data Type	Latency	Data Rate	Packet Loss Rate
Haptic feedback	3–5.5 ms	128–400 Kbps	<10 power-4
3D Camera Flow	<150 ms	137 Mbps–1.6 Gbps	<10 power-3
Physical vital signs	<250 ms	10 kbps–1.536 Mbps	<10 power-3

## Data Availability

Not Applicable.
